# Assessment of factors associated with PSA level in prostate cancer cases and controls from three geographical regions

**DOI:** 10.1038/s41598-021-04116-8

**Published:** 2022-01-07

**Authors:** Nishi Karunasinghe, Tsion Zewdu Minas, Bo-Ying Bao, Arier Lee, Alice Wang, Shuotun Zhu, Jonathan Masters, Megan Goudie, Shu-Pin Huang, Frank J. Jenkins, Lynnette R. Ferguson

**Affiliations:** 1 Auckland Cancer Society Research Centre, Faculty of Medical and Health Sciences (FMHS), University of Auckland, Private Bag 92019, Auckland, New Zealand; 2 Molecular Epidemiology Section, Laboratory of Human Carcinogenesis, National Cancer Institute, NIH, Bethesda, MD 20892 USA; 3 Department of Pharmacy, China Medical University, Taichung, 404 Taiwan; 4 Section of Epidemiology and Biostatistics, School of Population Health, University of Auckland, Private Bag 92019, Auckland, New Zealand; 5 Urology Department, Whangarei Hospital, Whangārei, New Zealand; 6 Urology Department, Auckland City Hospital, Auckland, New Zealand; 7 Department of Urology, Kaohsiung Medical University Hospital, Kaohsiung, 807 Taiwan; 8 Center for Cancer Research, Kaohsiung Medical University, Kaohsiung, 807 Taiwan; 9 Infectious Diseases and Microbiology and Clinical and Translational Science Institute, The University of Pittsburgh Medical Center, Hillman Cancer Center, Pittsburgh, PA USA; 10 Emeritus Professor, FMHS, University of Auckland, Private Bag 92019, Auckland, New Zealand

**Keywords:** Cancer, Genetics, Oncology, Risk factors, Urology

## Abstract

It is being debated whether prostate-specific antigen (PSA)-based screening effectively reduces prostate cancer mortality. Some of the uncertainty could be related to deficiencies in the age-based PSA cut-off thresholds used in screening. Current study considered 2779 men with prostate cancer and 1606 men without a cancer diagnosis, recruited for various studies in New Zealand, US, and Taiwan. Association of PSA with demographic, lifestyle, clinical characteristics (for cases), and the *aldo–keto reductase 1C3* (*AKR1C3*) rs12529 genetic polymorphisms were analysed using multiple linear regression and univariate modelling. Pooled multivariable analysis of cases showed that PSA was significantly associated with demographic, lifestyle, and clinical data with an interaction between ethnicity and age further modifying the association. Pooled multivariable analysis of controls data also showed that demographic and lifestyle are significantly associated with PSA level. Independent case and control analyses indicated that factors associated with PSA were specific for each cohort. Univariate analyses showed a significant age and PSA correlation among all cases and controls except for the US-European cases while genetic stratification in cases showed variability of correlation. Data suggests that unique PSA cut-off thresholds factorized with demographics, lifestyle and genetics may be more appropriate for prostate cancer screening.

## Introduction

The serine protease prostate-specific antigen (PSA) is encoded by the k*allikrein-related peptidase* 3 (*KLK3)* gene located in chromosomal region 19q13^1^. This protease is exclusively produced by the glandular prostate epithelium^2^. PSA reaches the serum when the microarchitecture of prostate glandular tissue is disrupted^3^. Accordingly, increased level of PSA in serum is an indication of prostate disease or trauma in the prostate gland including prostate cancer.

Since PSA was considered a marker for prostate cancer screening by the US Food and Drug Administration in 1994^4^, US health services had a dedicated prostate cancer screening until the year 2008, when prostate cancer screening with PSA reached a controversial status. This was due to the debate as to whether PSA based screening can help reduce prostate cancer-related mortality in men. Benefits of the PSA biomarker on reducing prostate cancer-related mortality estimated between the European Randomised Study of Screening for Prostate Cancer (ERSPC), and the US Prostate, Lung, Colorectal, and Ovarian Cancer (PLCO) trial varied, while both studies identified trial design shortcomings^5–8^. A recent analysis of both ERSPC and PLCO data using the cure parameter estimation calibrated to the ERSPC, projects comparable mortality reductions between the PLCO and the ERSPC trials^9^. In the year 2012, the US Preventive Services Task Force (USPSTF) advised against PSA-based screening for prostate cancer by issuing a grade D recommendation^10^. Comparison of prostate cancer diagnosis data during the pre- and post-USPSTF recommendation eras show that during the latter period US men were diagnosed with more advanced disease compared to the former^11^.

Meanwhile, various age-specific PSA thresholds for prostate cancer screening have been used or tested^12^. Literature suggests multiple other factors associated with PSA including BMI^13^, tobacco smoking^14–16^, alcohol consumption^17^, ethnicity^16^, and single nucleotide polymorphisms (SNPs) associated with both PSA alone or with PSA and prostate cancer^18–22^. Li et al. 2020 have recorded SNPs that alter functions of nearby genes leading to age-specific effects on PSA level^23^. Association of genetic polymorphisms in the superfamily of organic anion-transporting polypeptides, encoded by SLCO2B1 and SLCO1B3 genes with a potential to adrenal androgen transport to prostate cells have been shown to regulate PSA levels^24^. However, multiple variable factor assessments associated with PSA especially in cohorts from various continents are not found in the literature.

Meanwhile, SNPs associated with extra-testicular androgen production affecting PSA levels are limited. PSA production is signalled by the androgen activated androgen receptors^25^. Androgens are mainly produced in the testicular tissue involving the conversion of androstenedione to androgen catalysed by the *17β-hydroxysteroid dehydrogenase 3*^26^. The relevance of the *aldo–keto reductase 1C3* (*AKR1C3*) and its promoter gene polymorphisms in prostate cancer and its catalytic significance in producing extra-testicular androgens including the more potent dihydrotestosterone (DHT) are known^27,28^. A study involving New Zealand (NZ) men has shown that PSA level also has an association with the *AKR1C3 rs12529* genetic polymorphism^29^. Other *AKR1C3* genetic polymorphisms have also been associated with benign prostate hyperplasia-related factors including dihydrotestosterone inactivation and prostate volume^30^. Meanwhile, our data suggests that the age-based PSA variation is restricted to the *AKR1C3* rs12529 CG and GG genotype carriers in a NZ prostate cancer cohort^31^. In that study, we also showed that ever- smoking influenced both age and PSA at prostate cancer diagnosis among the *AKR1C3* rs12529 GG and CG genotype carriers, respectively.

The PSA cut-off thresholds required for prostate screening could differ among different populations and generalised thresholds can cause misjudgement. The current study therefore reports a cross-sectional analysis to understand PSA associations with demographic, lifestyle, and genetic factors (based on the *AKR1C3* rs12529 SNPs) in pooled as well as individual case and control cohorts from three geographical regions in the world.

## Material and methods

### Participant recruitment and data collection

Participants assessed in this study were those that were recruited for previous studies from the study locations. Data availability for various study cohorts therefore have similarities as well as differences as follows.

### NZ case control cohorts

Details of the NZ prostate cancer patient cohort (N = 515) considered in this analysis are described in detail elsewhere^31^. Patients were recruited between 2006 and 2013 with informed and signed consent (ethics reference NTY/05/06/037 from the Health and Disability Ethics Committees, Ministry of Health, NZ). Patient factors, including self-reported ethnicity, current/former tobacco smoking status and alcohol consumption were recorded at recruitment. Drinking one or more alcoholic drinks per week equivalent to a can of beer, a small glass of wine or a single nip (approximately 20 ml) of spirits categorized men as alcohol consumers. At recruitment, patient heights and weights were measured at the study centre for body mass index (BMI) estimation. Clinical and pathology records of patients were evaluated at the hospital databases to collect age, PSA level, Gleason score and disease stage [tumour-node-metastasis (TNM)] at diagnosis. In this study, patient risk (high or low) status was further stratified based on the disease prognostic stage grouping which followed the criteria defined by the 7^th^ edition of the AJCC abbreviated as I, IIA,IIB,III and IV as mentioned previously^32^.

All controls (n = 572) were NZ-Europeans, recruited for the 'Optimisation of selenium for health benefits' study from the Auckland region. This study is registered with the Australian New Zealand Clinical Trial Registry (ANZCTR)^33^. These men, self-reported as having no history of cancers (other than non-melanoma skin cancers), and not taking more than 50 µg selenium/day as supplements were recruited to this study^34^. Participant recruitment was carried out with informed and signed consent (ethics reference NTY/06/07/060, from the Health and Disability Ethics Committees, Ministry of Health, NZ). Recruitment of men to this study started in October 2006 and completed in December 2009. Height and weight of each participant was measured and recorded at study entry, for BMI estimation. These men were of the age range ≥ 20y to ≤ 80y. At study entry, participants completed a health and lifestyle questionnaire that provided information, including age at recruitment, tobacco smoking and alcohol consumption lifestyle. Alcohol consumer status was categorized similar to that of the NZ cases. Additionally, controls were to provide details of long-term medication used if any, and the disorder being treated. Based on the medication types used and the disorders being treated, they were categorised as having BPH or LUT, cardiovascular disease, diabetes, mental illnesses, or other medical conditions. Those not recording use of any medication and have not indicated a health disorder were considered as healthy controls.

### US case control cohorts

The US cohort is part of the NCI-Maryland Prostate Cancer Case–Control Study and has been described previously^35^. Recruitment was carried out between 2005 and 2015 under the ethics approval by the Institutional Review Boards at the NCI [protocol # 05-C-N021] and the University of Maryland [protocol #0298229]^35^. Of the 976 cases that were recruited into the study, 489 were African Americans (US-AA) and 487 were European Americans (US-EA). For the study herein, other patient clinical information (age at diagnosis, PSA at diagnosis, TNM stage and Gleason score at diagnosis) collected from pathology reports and medical records of 202 of these US-AA and 232 US-EA cases were also available for analysis. Disease prognostic stage grouping, and risk classification followed the criteria as mentioned before for NZ cases. 486 US-AA and 548 US-EA healthy controls within the age range 47 to 92 were also recruited for the controls arm of the study. All cases and controls self-reported to be either US-AA or US-EA at an interview and signed an informed consent to participate in the study. The interview also evaluated lifestyle factors that included tobacco-smoking habits and alcohol consumption. Alcohol consumers were considered as those consuming more than 12 alcoholic beverages per year, such as beer, wine, wine coolers or liquor. At recruitment, participants were asked their current heights and weights for BMI estimation.

### Taiwanese patient cohorts

645 patients with advanced prostate cancer who were on androgen-deprivation therapy (ADT) (TW1 cohort); and 643 patients with localized prostate cancer who underwent RP as initial treatment (TW2 cohort) were recruited between 1995 and 2009. Recruitments were made from three medical centres in Taiwan: Kaohsiung Medical University Hospital, Kaohsiung Veterans General Hospital, and National Taiwan University Hospital, as previously described^36,37^. According to these authors, all TW1 patients have been treated with ADT either with orchiectomy or with luteinizing hormone—releasing hormone agonist, with or without anti-androgen, and were prospectively monitored to evaluate the efficacy of ADT. TW2 patients were treated with RP as initial therapy for localized prostate cancer. Recruitment was carried out under the ethics approval by the Kaohsiung Medical University Hospital (IRB # KMUHIRB-2013132), with informed and signed consent of the patients. At recruitment, patient heights and weights were measured for BMI estimation. Tobacco smoking and alcohol consumption data was not available for these cohorts. Baseline clinical-pathological characteristics (age, PSA level, Gleason score and TNM stage at diagnosis) were collected from patient records. The TNM T2 sub-stage classifications (T2a, T2b and T2c) were not available for the Taiwan cohorts. Although prostate cancer risk status in TW men followed criteria mentioned before under NZ cases, a variation was employed to categorize men carrying TNM stage of T2. Therefore, patients recording a PSA at diagnosis < 20 ng/ml or a Gleason sum score < 8 with a ≤ T2 stage were considered as carrying the AJCC prognostic stage grouping < IIB (low-risk disease). TW men with a T3 or T4 stage, or PSA level of ≥ 20 ng/ml, or a Gleason sum score of ≥ 8 with stage 2 were considered as carrying the prognostic stage grouping ≥ IIB (high-risk disease).

### SNP genotyping

Genotype data for the TW1 cases cohorts, the NZ, and US cases cohorts and NZ controls cohort were accessed from previously published studies^29,32,36,38^. SNP genotyping of TW1, NZ and US cases and controls cohorts were performed using protocols described elsewhere^29,32,36^. Genotyping of the TW2 cases cohorts was performed using the Agena Bioscience MassArray iPLEX platform at the National Center for Genome Medicine, Academia Sinica, Taiwan.

### PSA measurements

The PSA measurement platforms used in the cases sample assays in NZ and US were not recorded at the time of the study and the historical information on methods used were accessed for the preparation of this manuscript. The pre-biopsy PSA testing on fresh serum samples of NZ cases were carried out at the community testing facilities managed by either the Diagnostic Medlabs, Auckland until 2009 or Labtests, Auckland since then using the ADVIA Centaur XP platform (Siemens Diagnostics), following the manufacturer’s protocols. There were times when PSA measurements were repeated prior to biopsy at the hospital laboratories that have used different measurement platforms including Modular E170 (Roche Diagnostics, NZ) from 2006–2012, and Cobas e602 (Roche Diagnostics, NZ) thereafter with the manufacturer’s protocols. Fresh serum samples of US cases were assessed using the VITROS total PSA II method with manufacturer’s protocols. Fresh serum samples of all TW cases cohorts were assayed with the Access Hybritech PSA assay method on the Access Immunoassay System (Beckman Coulter, Fullerton, CA, USA).

At recruitment, NZ and US controls provided blood samples in BD plain vacutainer tubes. For serum separation, the NZ samples were spun at 2000 g for 10 min on an Eppendorf 5810R centrifuge (Hamburg, Germany), while the US samples were spun at 850 g for 10 min on a Sorvall T 6000 (Thermo Scientific, New Jersey, USA).

Serum aliquots were stored in − 80 °C until PSA measurements were made. Storage time for NZ controls samples prior to PSA measurements ranged from 3–6 y while that of US controls samples ranged from 3–13 y. The total PSA was measured from stored NZ serum aliquots at LabPlus, Auckland, NZ using electro-chemiluminescence immunoassay (Roche Cat. #. 04641655 190) on a Roche Modular E170 analyzer (Roche Diagnostics, NZ). Total assay imprecision was 3.2% at a level of 1.12 ng/mL, 3.7% at 4.61 ng/mL, and 2.7% at 27.5 ng/ml. Serum PSA measurements of US-AA and US-EA controls were obtained using the Human Total Prostate Specific Antigen ELISA Kit from AbCam, ab188388 (Cambridge, United Kingdom). Each sample was measured in duplicate. The average % coefficient of variance was 8.67.

### Statistical analysis

The NZ prostate cancer cases cohort consisting of self-identifications exclusive for Māori, Pacific-Peoples and East-Asian (n = 17) were aggregated as one group (NZ-MPEA) due to their high *AKR1C3* rs12529 G allele frequency (85%) recorded in this study. This frequency is comparable to that of the current Taiwan cohort (88%), as well as the East-Asian cohorts reported previously (86%)^39^. The rest of the NZ prostate cancer cohort was aggregated as NZ- non-MPEA group and consisted of New Zealanders self-identified as European, and those from the Indian sub-continent, Middle-East and others (n = 498). Participants with current and former tobacco smoking lifestyles were categorized as ever-smokers while the others were considered as never-smokers, without a set threshold. Continuous demographic variables were compared using the Kruskal–Wallis One Way Analysis of Variance on Ranks test, as most data types were not normally distributed. Measurements for non-normally distributed data were provided as medians and 25% and 75% inter quartile ranges. Categorical variables were tested with the Chi Square test. Combined overall PSA data were found to be highly right skewed. Therefore, for the subsequent multiple linear regression analyses, and univariate analyses, PSA data were log transformed. Multiple linear regression analysis was carried out to test the association of PSA with ethnicity, BMI, tobacco smoking, alcohol consumption status, age at recruitment (for controls), age at diagnosis (for cases), disease prognostic stage and Gleason sum score (for cases) and the *AKR1C3* rs12529 genotype, as well as for analysing interaction effects. The Spearman Rank Order Correlation was used to analyse the correlation between age and log PSA for all NZ- non- MPEA, US-EA, US AA, TW1 and TW2 cases cohorts and the available controls cohorts with and without genetic stratification. As the NZ- MPEA group consisted of only 17 individuals, they were excluded from all Multiple linear regression and Univariate analyses. Statistical analyses were performed using SAS 9.4 (SAS Institute Inc., Cary, NC, USA) and SigmaPlot version 14.0 (Systat Software Inc.). A two-sided significance level of P < 0.05 was set out for all analyses. Violin plots, histograms and correlation plots were created in R (version 4.1)^40^ using the ggplot2 package^41^.

## Results

### Characteristics of the prostate cancer cohorts

A comparison of overall patient characteristics (demographic, lifestyle, and clinical) is given in Supplementary results Table S1a. The Taiwan (TW1-advanced, and TW2-localized prostate cancer groups respectively) cases showed a significantly lower median BMI (24.2 kg/m^2^ and 24.7 kg/m^2^ respectively) compared to other cases (27.0 kg/m^2^ for NZ, 27.7 kg/m^2^ for US-AA and 27.5 kg/m^2^ for US-EA and P < 0.001). The US-AA cases recorded a significantly higher percentage of ever-smokers compared to NZ and US-EA cases (72% vs 56% for NZ and 61% for US-EA and P < 0.00001). The percentage alcohol consumption among NZ cases were significantly lower than the US-AA and the US-EA cases (71% vs 85% for US-AA and 90% for US-EA and P < 0.00001). Lifestyle data related to tobacco smoking and alcohol consumption are not available for TW1 and TW2 cases.

**Figure 1:**
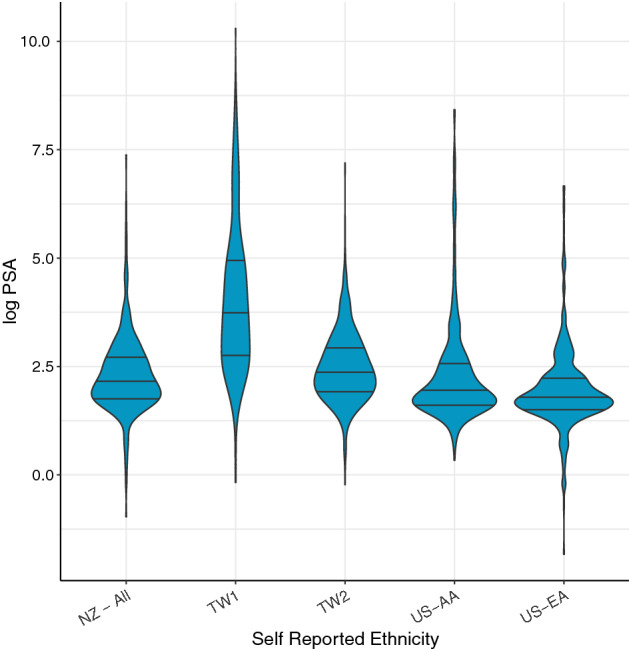
The probability densities of log PSA data of NZ-all, US-AA, US-EA, TW1 and TW2 cases. All violin plots are provided with the median and inter-quartile ranges. NZ-all = All NZ cases consisting of 94.2% European, 3.3%-Māori, Pacific and East Asian (MPEA); 2.5% from the Indian sub-continent and Middle-Eastern and others. US-AA = African American cases cohort. US-EA = European American cases cohort. TW1 = Taiwanese cohort with advanced prostate cancer who were on androgen-deprivation therapy. TW2 = Taiwanese cohort with localized prostate cancer who underwent RP as initial treatment.

**Figure 2:**
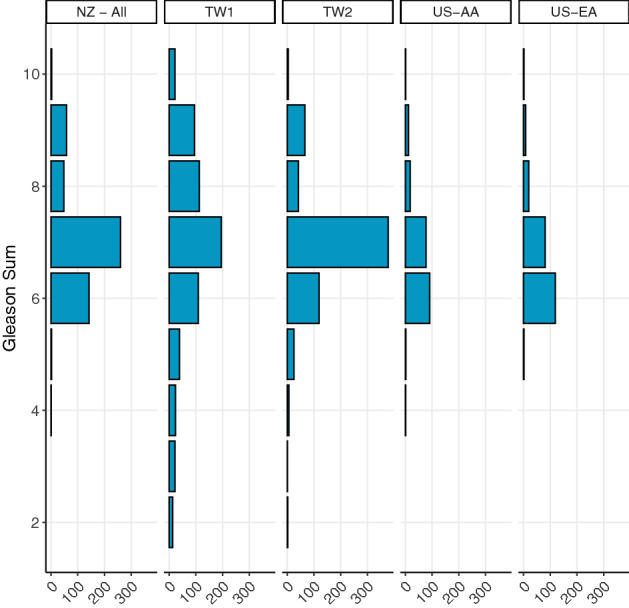
Distribution of the Gleason sum scores of NZ-all, US-AA, US-EA, TW1 and TW2 cases. NZ-all = All NZ cases consisting of 94.2% European, 3.3%-Māori, Pacific and East Asian (MPEA); 2.5% from the Indian sub-continent and Middle-Eastern and others. US-AA = African American cases cohort. US-EA = European American cases cohort. TW1 = Taiwanese cohort with advanced prostate cancer who were on androgen-deprivation therapy. TW2 = Taiwanese cohort with localized prostate cancer who underwent RP as initial treatment.

The probability densities of log PSA and the Gleason sum score of cases between different ethnic groups are given in Figs. 1 and 2 respectively while median values of clinical data are given in Supplementary results Table S1a. Median PSA at prostate cancer diagnosis was higher among the TW1 and TW2 cases (41 ng/ml and 11 ng/ml respectively vs 8.6 ng/ml for NZ, 7.0 ng/ml for US-AA and 5.8 ng/ml for US-EA and P < 0.001). Median Gleason sum score was the lowest among US-EA cases (6 vs 7 for the NZ, TW1, TW2, and US-AA cases; P < 0.001). TW cases recorded a relatively higher proportion of Gleason sum score ≤ 5 cancers compared to other cohorts. A significantly higher percentage of high-risk prostate cancer with a prognostic stage of  ≥ IIB (86%) was recorded among the TW1 cases compared to 29–66% among other cases (TW2, NZ, US-AA, and US-EA) (P < 0.00001). Median age at prostate cancer diagnosis was significantly higher among TW1 cases compared to NZ, TW2, US-AA, and US-EA cases (73 y vs 66 y each for NZ and TW2, 63 y for US-AA, and 65 y for US-EA and P < 0.001).

**Figure 3:**
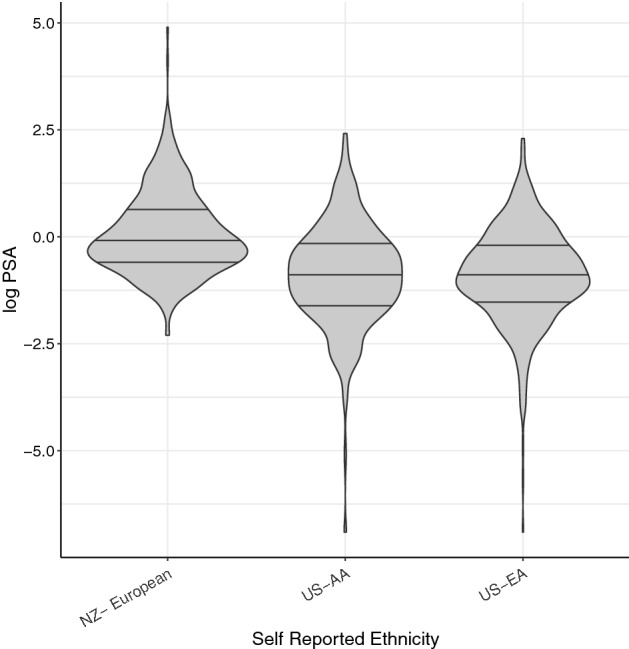
The probability densities of log PSA data of NZ-European, US-AA and US-EA controls. All violin plots are provided with the median and inter-quartile ranges. NZ-European controls = NZ controls cohort with self-reported European ethnicity. US-AA = African American controls cohort. US-EA = European American controls cohort.

A comparison of overall controls characteristics (demographic, lifestyle, and PSA) is given in Supplementary results Table S1b. The probability densities of log PSA of controls between different ethnic groups are given in Fig. 3. Median PSA at recruitment was significantly different between the three controls cohorts (NZ-0.9 ng/ml, US-AA-0.4 ng/ml and US-EA-0.4 ng/ml). The NZ controls were significantly younger (median 54 y) than the controls from the US (US-AA median 66 y and US-EA median 64 y, < 0.001). Significantly, different BMI values were recorded between the three cohorts with US-AA recording the highest median of 29 kg/m^2^ and NZ and US-EA controls recording medians of 26 and 27.4 kg/m^2^ respectively. Significantly different proportions of ever-smokers were recorded between the three controls cohorts with only 34% among NZ controls while US-AA and US-EA controls recorded 61% and 59% respectively. Among NZ controls, 60.8% have recorded no medication intake for any health disorder, while 21% were taking medication for cardiovascular disease, 1% for diabetes, 5.8% for benign prostatic hyperplasia / lower urinary tract infection (BPH/LUT), 3.7% for mental illnesses, and 8.2% for other health disorders. Such data is not available for the US control cohorts.

### *AKR1C3* rs12529 genetic polymorphism distribution among the cohorts

The *AKR1C3* rs12529 genotype data for a total of 366, 202, 232, 618 and 643 of cases from NZ-non-MPEA, US-AA, US-EA, TW1 and TW2 cohorts respectively, 13 NZ-MPEA cases and 454 from NZ controls are presented in Supplementary results Table S2. The NZ-MPEA and TW cases recorded a frequency of the *AKR1C3* rs12529 G allele between 84%-88%, while in NZ- non-MPEA, US-AA and US-EA cases this was 45%. NZ-European controls recorded a frequency of 38% for the *AKR1C3* rs12529 G allele.

### Multiple variable testing on log PSA outcomes

I. Testing the association of log PSA on multiple variables among pooled cases.

**I.a.** A summary of the association of log PSA with ethnicity, disease prognostic stage, Gleason sum score, age at diagnosis, BMI and genotype for cases cohorts analysed with or without lifestyle factors is given in Table 1. Log PSA showed a significant association with all tested factors except for the genotype when analysed without lifestyle factors. The US-AA, NZ-non-MPEA, TW1 and TW2 cases cohorts showed a higher log PSA compared to that of the US-EA cohort. BMI showed a significant negative association on log PSA while the other variables showed a positive association.

**Table 1:** Results summary of multiple linear regression analyses for testing impacts of demographic, genetic, lifestyle and clinical parameters on log PSA for all cases cohorts.

Without lifestyle* data			With lifestyle* data		
Parameter	Parameter Est.	Pr > F	Parameter	Parameter Est.	Pr > F
Ethnicity (ref = European American)		< .0001	Ethnicity (ref = European American)		0.0002
African American	0.35		African American	0.36	
NZ-non MPEA	0.29		NZ-non MPEA	0.24	
Taiwanese TW1	1.69				
Taiwanese TW2	0.51				
Prognostic Stage (ref = < IIB)		< .0001	Prognostic Stage (ref = < IIB)		< .0001
≥ IIB	0.55		≥ IIB	0.33	
Gleason sum score	0.18	< .0001	Gleason sum score	0.27	< .0001
Genotype (ref = CC)		0.678	Genotype (ref = CC)		0.653
CG	− 0.02		CG	− 0.006	
GG	0.04		GG	0.069	
Age at diagnosis	0.01	0.0004	Age at diagnosis	0.01	0.003
BMI	− 0.02	0.004	BMI	− 0.01	0.136
			Ever-smoker (ref = never smoker)	0.16	0.015
			Alcohol consumer (ref = never alcohol consumer)	− 0.07	0.370
Model	R^2 = 0.396, Pr > F < 0.0001		Model	R^2 = 0.187, Pr > F < 0.0001	

Ever-smoker and alcohol consumer lifestyle data are not available for Taiwanese (TW) cohorts.

NZ-non MPEA cases – New Zealanders self-identified as European, or from the Indian sub-continent, Middle-Eastern and others.

When lifestyle factors of tobacco smoking and alcohol consumption were included in the model, log PSA showed a significant association with ethnicity, disease prognostic stage, Gleason sum score, age at diagnosis and smoking status. In this analysis too, genotype showed no association on log PSA. Alcohol consumption also showed no significant association with log PSA outcomes. The log PSA association with BMI was not significant in this analysis compared to the analysis which included TW cases, but without inclusion of lifestyle data.

**I.b.** Interactive effects on log PSA.

Multiple linear regression for the interactions between age at diagnosis, lifestyle, genetics, and ethnicity in the log PSA outcomes were analysed. However, except for the age at diagnosis and ethnicity two-way interaction (Table 2), the interactions between ethnicity and ever-smoking status or ethnicity and alcohol consumption status or the three-way interaction between age at diagnosis, ethnicity and genotype were not significantly associated with log PSA (Supplementary results Tables S3). However, the age at diagnosis*ethnicity interaction remained significant even under the three-way model.

**Table 2:** Statistical outcomes in the interactive model with age at diagnosis and ethnicity on log PSA outcome for US-EA, US-AA, and NZ-non-MPEA cases cohorts.

Source	DF	Type III SS	Mean Square	F value	Pr > F
Age at diagnosis*ethnicity interaction					
Ethnic group	2	5.38	2.69	3.33	0.036
Prognostic stage	1	17.43	17.43	21.56	< .0001
Gleason sum score	1	36.93	36.93	45.69	< .0001
Genotype	2	0.66	0.33	0.41	0.667
Age at diagnosis	1	7.51	7.51	9.28	0.002
BMI	1	1.85	1.85	2.29	0.130
Smoker	1	4.99	4.99	6.17	0.013
Alcohol	1	0.60	0.60	0.75	0.388
Age at diagnosis*Ethnic Group	2	8.08	4.04	5	0.007

NZ-non MPEA cases – New Zealanders self-identified as European, Indian sub-continent, Middle-East and others.

II. The association of log PSA with multiple variables in independent cases cohorts.

As cases data showed a significant interaction of age at diagnosis and ethnicity with log PSA outcomes, all cases cohorts were also analysed independently with multiple linear regression. Independent cases cohorts assessed with multiple linear regression analysis (Table 3) indicate that log PSA is significantly associated with Gleason sum score for US-EA cases; Gleason sum score and BMI for US-AA cases; prognostic stage, age at diagnosis and tobacco smoking for NZ-non-MPEA cases; prognostic stage and BMI for TW1 cases and prognostic stage and Gleason sum score for TW2 cases.

III. The association of log PSA with multiple variables among pooled controls.

**Table 3:** The association of log PSA with age, BMI, clinical data, lifestyle and genotype for US-EA, US-AA and NZ-non-MPEA cases cohorts analysed independently.

Parameter	European American		African American		NZ-non-MPEA		TW1		TW2	
	Estimate	Pr > F	Estimate	Pr > F	Estimate	Pr > F	Estimate	Pr > F	Estimate	Pr > F
Prognostic Stage ≥ IIB (ref = < IIB)	0.13	0.310	0.22	0.179	0.62	< .0001	1.79	< .0001	0.35	0.0003
Gleason sum score	0.26	0.001	0.62	< .0001	0.07	0.208	0.08	0.098	0.12	0.006
Genotype (ref = CC) for all except TW (ref = CG)		0.361		0.720		0.778				
CG	0.08		− 0.047		− 0.07		0.00	0.117	0.00	0.666
GG	0.024		0.105		− 0.01		− 2.35		0.44	
Age at diagnosis	0.01	0.491	0.02	0.073	0.02	0.0003	− 0.02	0.894	0.01	0.186
BMI	− 0.01	0.587	− 0.04	0.012	0.01	0.259	− 0.06	0.006	0.01	0.659
Ever-smoker (ref = never smoker)	0.16	0.697	0.13	0.697	0.22	0.018				
Alcohol consumer (ref = never alcohol consumer)	− 0.07	0.595	0.21	0.325	− 0.15	0.139				
Model	R^2 = 0.08, Pr > F < 0.013		R^2 = 0.336, Pr > F < 0.0001		R^2 = 0.206, Pr > F < 0.0001		R^2 = 0.23, Pr > F < 0.0001		R^2 = 0.111, Pr > F < 0.0001	

NZ-non MPEA cases – New Zealanders self-identified as European or from the Indian sub-continent, Middle-East and others.

Multiple regression analysis showed that log PSA is significantly associated with ethnicity, age, BMI, and smoking status when all controls cohorts were considered together (Table 4).

- IV. The association of log PSA with multiple variables in independent controls cohorts.

**Table 4:** Results of multiple linear regression analysis for testing impacts of age, BMI, and lifestyle on log PSA for all controls cohorts.

Parameter	Parameter Est.	Pr > F
Ethnicity (ref = NZ-European)		< 0.0001
African American	− 1.27	
European American	− 1.41	
Age	0.04	< .0001
BMI	− 0.02	0.0002
Ever-smoker (ref = never-smoker)	− 0.12	0.036
Ever-alcohol consumer (ref = never-alcohol consumer)	− 0.003	0.965
Model	R^2 = 275, Pr > F < 0.0001	

When the controls cohorts were independently analysed with multiple linear regression analysis (Table 5), age was significantly associated with log PSA in US-EA, US-AA, and NZ controls. However, in US-EA and US-AA controls, log PSA was significantly associated also with BMI, while among US-AA controls, tobacco smoking was also a significant association factor.

**Table 5:** Summary of multiple linear regression analysis for testing impacts of age, BMI, and lifestyle on log PSA for US-EA, US-AA and NZ-European controls analysed independently.

Parameter	European American		African American		NZ-European	
	Parameter Est.	Pr > F	Parameter Est.	Pr > F	Parameter Est.	Pr > F
Age	0.03	< .0001	0.05	< .0001	0.03	< .0001
BMI	− 0.02	0.048	− 0.03	0.008	− 0.01	0.2039
Ever-smoker (ref = never-smoker)	− 0.18	0.072	− 0.25	0.034	0.04	0.6255
Ever alcohol consumer (ref = never alcohol consumer)	0.13	0.389	0.09	0.4812	− 0.19	0.0785
Model	R^2 = 0.064, Pr > F < 0.0001		R^2 = 0.13, Pr > F < 0.0001		R^2 = 0.282, Pr > F < 0.0001	

### Univariate analyses on log PSA correlation with age

**Figure 4:**
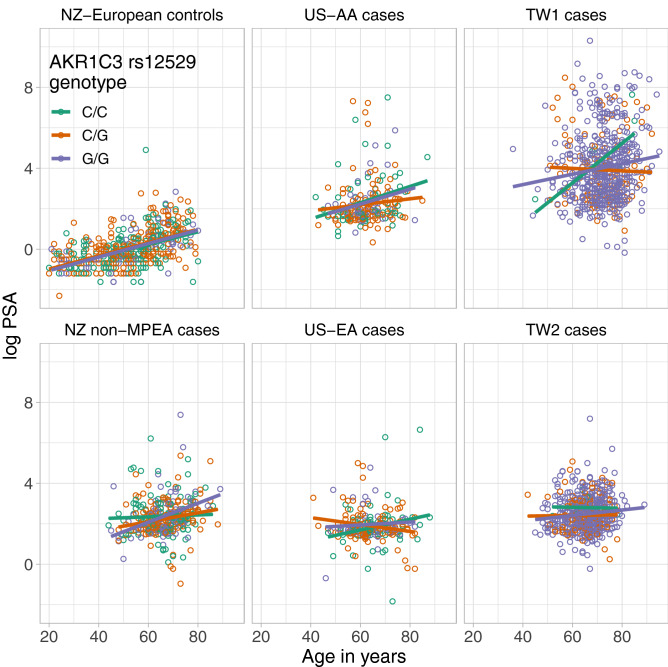
Correlation scatter plots between age and log PSA at recruitment for NZ-European controls and age and log PSA at diagnosis for NZ-non-MPEA, US-AA, US-EA, TW1 and TW2 cases stratified by the *AKR1C3* rs12529 genotypes. All plots are presented with linear trend lines. NZ-European controls = NZ controls cohort with self-reported European ethnicity. NZ-non-MPEA cases = New Zealand cases self-identified as European or from the Indian sub-continent, Middle-East and others. US-AA = African Americans cases cohort. US-EA = European Americans cases cohort. TW1 = Taiwanese cases cohort with advanced prostate cancer who were on androgen-deprivation therapy. TW2 = Taiwanese cases cohort with localized prostate cancer who underwent RP as initial treatment.

As ethnicity interacting with age was the most influential factor that produced an impact on log PSA, we further attempted univariate analyses to understand age dependent impacts on log PSA levels with and without stratification by genotype for independent case and control cohorts (Table 6). Correlation scatter plots between age and log PSA for NZ-European controls and age at diagnosis and log PSA for NZ-non-MPEA, US-AA, US-EA, TW1 and TW2 cases with stratification by the *AKR1C3* rs12529 genotypes are given in Fig. 4 with linear trend lines. The *AKR1C3* rs12529 CC genotype carriers were poorly represented in TW1 (n = 8) and TW2 (n = 6) cases cohorts. Overall, all controls (NZ, US-EA, US-AA) and all cases except for the US-EA cases showed significant correlation between age and log PSA. A reduction in correlation coefficient strength was observed among cases compared to controls overall. The NZ control cohort showed significant age and log PSA correlation despite stratification by genotype. However, NZ-non-MPEA cases showed significant age and log PSA correlation only among those carrying the *AKR1C3* rs12529 CG and GG genotypes. For US-AA cases, significant age and log PSA correlation was restricted to those carrying the *AKR1C3* rs12529 CC and CG genotypes. For TW1 and TW2 cases, significant correlation was restricted to men carrying the *AKR1C3* rs12529 GG genotype, while for the US-EA cases, none of the *AKR1C3* rs12529 genotypes showed significant correlations.

**Table 6:** Spearman correlation statistics between age (age at diagnosis for cases and age at recruitment for controls) and log PSA stratified by ethnicity, case, control status and the *AKR1C3* rs12529 genotype.

	All	CC	CG	GG
**NZ European controls**				
r	0.556	0.517	0.519	0.616
p	2E−07	2E−07	2E−07	2.26E−09
n	498	181	202	71
**NZ-non MPEA cases**				
r	0.303	0.129	0.287	0.426
p	6.67E−011	0.160	2.33E−04	7.43E−05
n	449	120	161	82
**US-AA controls**				
r	0.344			
p	1.09E−12			
n	410			
**US-AA cases**				
r	0.243	0.312	0.239	0.153
p	4.98E−04	0.017	0.015	0.349
n	202	58	105	39
**US-EA controls**				
r	0.213			
p	2.89E−06			
n	475			
**US-EA cases**				
r	0.0244	0.113	− 0.063	0.110
p	0.711	0.352	0.504	0.457
n	232	69	115	48
**TW1 cases**				
r	0.119	0.500	− 0.00108	0.140
p	0.003	0.182	0.990	0.002
n	622	8	133	477
**TW2 cases**				
r	0.113	0.0286	0.103	0.121
p	0.005	1.000	0.217	0.008
n	622	6	144	472

r = correlation coefficient; p = significance of probability; n = number of pairs tested.

NZ-non MPEA cases – New Zealanders self-identified as European, or from the Indian sub-continent, Middle-Eastern and others.

## Discussion

The current analyses attempted to utilize available data from prostate cancer case control cohorts from three geographical regions to understand factors associated with PSA level. All tested characteristics between these cohorts were significantly different except for the *AKR1C3* rs12529 genetic polymorphisms that were clustered in to two groups. Among cases we observed a lower frequency (45%) of the *AKR1C3* rs12529 G allele in NZ- non-MPEA, US-AA and US-EA cohorts and a higher frequency (83–88%) represented by NZ-MPEA and TW cases cohorts respectively. Higher frequency of the *AKR1C3* rs12529 G allele recorded in the current TW and NZ-MPEA cohorts are comparable to recorded frequencies for the Asians^39^.

The current ever- smoker proportion of men among US-AA and US-EA cases and controls are higher than the proportions recorded during 2001–2012 period by Murphy et al.^42^. The current NZ cases cohort had a higher (56%) and the current NZ controls recorded a lower (34%) proportion of ever-smokers than 2011/2012 cohorts reported before^43^. The percentage of men recording alcohol consumption between NZ, US-AA, and US-EA cohorts (both cases and controls) were significantly different. However, due to the variation of alcohol consumption criteria between NZ and US cohorts, comparison of these statistics is not possible.

A Taiwanese study that evaluated prostate biopsies made during 1994–2003 records that the Gleason score ≤ 6 cancer detections have increased from 16.6 to 40.1% during the last five years^44^. Therefore, the relatively higher proportion of cancers assigned to a Gleason score of ≤ 5 in current TW cases compared to other cases cohorts could be due to a general trend in pathology reporting in TW. The TW1 cohort recorded the highest proportion (86%) of high-risk prostate cancers as expected, as this group was particularly recruited as an advanced prostate cancer category. The proportion of high-risk prostate cancers between NZ (51%) and US-EA (54%) cohorts were comparable; while the comparatively higher proportion of 66% high-risk prostate cancers recorded from the US-AA cohort was as expected^45^. The comparatively lower proportion of 29% of high-risk cases recorded from TW2 is due to recruitment of cases to this cohort particularly with localized prostate cancer category.

Sample types available for the serum PSA measurements between cases and controls differed by way of fresh samples for cases and samples stored at -80 °C for 3–6 y for NZ controls and 3–13 y for US controls. According to Scaramuzzino et al.2007, PSA protein is stable in − 70 °C at least for five years^46^. Simanek et al.reports a 3.6% decline in total PSA after 10 y storage in − 80 °C^47^. The total PSA measurements made in controls serum and plasma samples stored in − 20 °C for a median of 20 y in comparison to measurements made in comparable age-matched controls cohorts from the same era of sample collection have indicated the stability of this analyte after long-term storage^48^. Therefore, the current PSA readings made between controls samples stored for varying durations at − 80 °C could not have impacted the recordings and represent *in-situ* status of PSA levels.

Data collected from men with no known prostate cancer or BPH available in the Electronic Medical Records and Genomics (eMERGE) Network study (2015–2020) and for men without prostate cancer recorded in the pre- or early PSA era in the Malmö Diet and Cancer (MDC) study (1991–1996) have recorded median PSA levels and ages of 0.67 ng/ml and 56 y & and 1.12 ng/ml and 62y respectively^23^. The current median PSA levels recorded for NZ-European controls with comparable ages are higher than the eMerge study and lower than the records of MDC study represented by a relatively older group of men. The median PSA levels recorded for US controls are lower than both the eMERGE and MDC studies. A previous study with US-AA controls between ages 40–79 y without prostate cancer recruited between mid-late 1990s has recorded a median PSA of 0.83 ng/ml^49^. A predominantly US-EA controls cohort from the Physicians’ Health Study recruited in the pre-PSA era 1982–1984, within the age range 40–59 y, median PSA ranged from 0.89 to 1.04 ng/ml^50^. The relatively higher PSA levels previously recorded for US-EA and US-AA controls either in the pre- or early PSA era being comparable to the PSA level of the current NZ-European controls may be a consequence of low level of PSA-based prostate cancer screening and subsequent procedures in prostate cancer diagnosis in NZ as recorded previously^32,51^.

Our multiple linear regression model that included prostate cancer cases from multiple ethnic groups indicated that log PSA is directly associated with ethnicity, age at diagnosis and clinical factors and inversely with BMI, while the genotype showed no effect. It is known that Gleason grading system is the most powerful prognostic predictor of prostate cancer^52^. As we have used two highly correlated variables of Gleason sum score and prognostic stage among the independent variables in our multiple regression analysis, that could have caused a multicolinearity issue^53,54^, supressing the strength of the genetic influence.

When lifestyle factors were included in the model using the cases cohorts from US-EA, US-AA and NZ-non-MPEA, log PSA was shown to be directly associated with ethnicity, age at diagnosis, clinical factors, and tobacco smoking, but not with BMI. This may be due to sample size reduction after TW cases elimination reducing statistical power. In the independent cohort analyses of cases data, we noted that parameters that significantly associate with log PSA varied with each cases cohort. US-EA and US-AA cases showed log PSA association with Gleason sum score. For the US-AA cases represented by 66% high-risk cases, and TW1 cases represented by 86% high-risk cases, BMI was a significant inverse factor for log PSA outcomes. For NZ-non-MPEA cases, disease prognostic stage, age at diagnosis and tobacco smoking had significant association with log PSA level. Impact of tobacco smoking on PSA in NZ men have been reported before^29^. The inverse association of log PSA with tobacco smoking seen in the current combine controls as well as independent US-AA controls recorded in our analyses is comparable with previous studies^16^. Contradictory nature of tobacco smoking impacts on log PSA among cases and controls in the current assessment require explanation. One possibility is that within a tumour environment, controlling further tissue damage caused by tobacco smoke constituents could be restrictive leading to increased leaching of PSA to the circulation. NZ men smoke cigarettes containing an unusually high content of polycyclic aromatic hydrocarbons (PAHs) and nicotine^55^. Influence of PAHs and its metabolism by AKR1C3 producing reactive metabolites such as O-quinones and subsequent oxidative DNA damage, DNA adduct formation, DNA lesions and mutations have been reviewed before^56^. Therefore, the positive association of log PSA on ever-smoking in NZ-non-MPEA cases may at least partially, be due to this exposure to high PAH content. As *AKR1C3* enzyme catalyses both extra-testicular androgen synthesis and metabolism of PAHs derived from tobacco smoke exposure^27^, negative association of log PSA with tobacco smoking is also a possibility if the PAH metabolism is given priority over androgen synthesis as seen among pooled controls as well as US-AA controls. 70% of US-AA smokers are known to use menthol cigarettes compared to 30% of US-EA smokers^57^. In vitro studies with tobacco (TFeL) and menthol (MFeL) flavoured e-liquids have shown 843 and 589 differentially regulated genes. MFeL have affected several pathways including metabolic pathways as well as steroid hormone biosynthesis, including the AKR1C1, AKR1C2 and AKR1C3 genes^58^. The authors have also shown that the MFeL increased AKR1C1 gene expression by 7.4-fold compared to AKR1C1 gene expression increase of 4.9-fold by TFeL. The AKR1C1 and AKR1C2 genes are known to reduce the more potent androgen, the DHT to 5α-androstane-3α,17β-diol (3α-diol) and 5α-androstane-3β,17β-diol (3β-diol), respectively^28^. This DHT metabolism by menthol containing cigarette smoking could be among the reasons for the significant reduction in PSA signalling among US-AA ever-tobacco smoker controls. Such reduction in PSA could even impair the PSA-based screen detection of prostate cancer among US-AA ever-tobacco smokers and these men may require a reduced PSA threshold for screening^59^.

It is interesting to note that the US-EA and US-AA cases cohorts who were diagnosed at a relatively lower median PSA level than the NZ-non-MPEA cases, log PSA was associated with Gleason sum score, while the NZ-non-MPEA and the TW1 cases diagnosed with relatively higher PSA levels, log PSA was associated with the prognostic stage. The TW2 cases, although diagnosed with a relatively higher PSA level than the US-EA, US-AA and NZ-non-MPEA cases, and carrying only 29% of high-risk cases, log PSA was associated with both Gleason sum score and prognostic stage. Concordance of 52% between Gleason scores recorded at biopsy and post-RP has been reported before, as the latter procedure provides a more accurate recording^60^. According to these authors, in men with PSA at diagnoses < 10 ng/ml the Gleason score concordance is better (61%) than those with PSA > 10 ng/ml (23%). As the current US cases showed a median PSA at diagnoses between 6–7 ng/ml, their PSA association with Gleason sum score could be stronger due to more accurate Gleason grading. With TW1 cases with a median PSA of 41 ng/ml, there was no significant association between PSA and Gleason sum score while PSA was significantly associated with prognostic stage. In the TW2 cohort with a median PSA of 10.9 ng/ml, there was still a significant association between PSA and Gleason sum score while also recording an association with prognostic stage. Although the NZ cases recorded a median PSA of 8.6 ng/ml, they recorded no PSA association with Gleason sum score, but instead was associated with prognostic stage. The above may indicate that the NZ-non-MPEA cases have a late diagnosis similar to the TW1 cases. In these multivariable linear regression models, log PSA was not associated with age at diagnosis in US-EA, US-AA, TW1 and TW2 cases cohorts when analysed independently. Whether it is a true effect or due to reduced sample sizes when independent analyses were performed is hard to infer.

When controls from US-EA, US-AA, and NZ-Europeans were analysed together using multiple linear regression, log PSA was significantly associated with ethnicity, age, BMI, and tobacco smoking lifestyle. However, when the controls cohorts were independently analysed, US-AA controls retained the significant log PSA association with age, BMI, and tobacco smoking, while in US-EA, this was limited to age and BMI and for NZ-European controls this was limited to age only. Significant inverse log PSA association with BMI in US-AA cases and US-EA and US-AA controls could be indicating a possibility of BMI being indirectly involved in prostate health in US cohorts. It is also known that US-AA men present more aggressive prostate cancers than the US-EA men^61^. The average baseline BMI of predominantly US-EA men with a mean age of 65 y, in 1993–1996 was 26 kg/m^2^^62^. Among these men those who remained prostate cancer free were with baseline BMI ranging from 25.5- 25.9 kg/m^2^ while those who developed advanced prostate cases were with a baseline of 25.8 kg/m^2^^62^. Comparison of current data with that of the mid 1990s, there is an increase in BMI by around 1.5 kg/m^2^ among US-EA cases and controls. Among the current NZ-non-MPEA cases cohort, median BMI was 1 kg/m^2^ higher than that of the current NZ-European controls cohort. As BMI is significantly and inversely associated with log PSA levels in some of the analysed cohorts, there is a possibility of higher BMI masking early diagnosis of prostate cancer by PSA screening in these cohorts^63^. Log PSA was significantly and inversely associated with BMI also in the TW1 cases cohort with advanced stages of prostate cancer receiving ADT. This BMI association with log PSA might have some relevance to ADT associated BMI increases in this cohort^64^. It is a possibility that BMI and associated inverse log PSA is unique to certain populations such as the current US-AA cases and US-AA and US-EA controls, indicative of a unique aetiology towards prostate health outcomes in these men. At the time the US Food and Drug Administration approved PSA to be used for prostate cancer screening in 1994^65^, predominantly US-EA men were with a mean BMI of 26 kg/m^2^^62^. It is possible that in mid 1990s age-based increase in PSA was a valid concept in US prostate cancer cohorts, although not so in the recent prostate cancer study cohorts from US with relatively higher BMI. A study of Asian men with a mean (range) BMI of 24.3 (13.5–34.8) kg/m^2^, PSA was inversely associated with BMI, however, increased BMI has not negatively influenced PSA accuracy for predicting prostate cancer^66^. However, these authors and others^67,68^ suggest that in prostate cancer screening, alternate PSA thresholds should be used in obese men.

All controls and prostate cancer cases except for the US-EA cases showed a significant correlation between age (at diagnosis for cases and at recruitment for controls) and log PSA based on a univariate analysis model. Compared to controls, cases showed a weakened but significant correlation in NZ-non-MPEA, and US-AA men while in US-EA cases this correlation was completely lost. It is known that serum PSA increase is not a specific marker for prostate cancer as it also increases with conditions such as BPH^18^. As the NZ controls consisted of only 5.8% of men with BPH/LUT, the age-associated increase in serum PSA in this cohort can be assured as predominantly due to age rather than due to urological disorders.

Our previous studies have reported that men carrying the *AKR1C3* rs12529 CC genotype carry an increased recording of high-risk or advanced prostate cancer in NZ and TW men^36,38^. Regardless of stratification by the *AKR1C3* rs12529 genetic polymorphism, NZ-European controls and NZ-non-MPEA cases carrying the *AKR1C3* rs12529 CG and GG genotypes retained a significant correlation between age at diagnosis and log PSA, while this was lost in NZ-non-MPEA cases carrying the CC genotype. Among both NZ-non-MPEA cases and NZ- European controls, the strongest correlation between age and log PSA was recorded among those carrying the *AKR1C3* rs12529 GG genotype. Among TW1 and TW2 cases cohorts, genetic stratification resulted in those carrying the *AKR1C3* rs12529 GG genotype retaining a significant correlation between the age at diagnosis and log PSA. Therefore, it seems that patients carrying the *AKR1C3* rs12529 CC genotype from NZ-non-MPEA, and CC and CG genotype carriers of TW, GG genotype carriers of US-AA and all US-EA cases carry prostate cancer phenotypes, without an association with age at diagnosis and PSA. It is interesting to note that the TW1 cases carrying the *AKR1C3* rs12529 GG genotype carry a significant age at diagnosis and log PSA correlation similar to the NZ-non-MPEA cases although the former has been diagnosed at a significantly higher age, PSA level, and prognostic stage while also recording a significantly lower BMI. Even the TW2 cases with the *AKR1C3* rs12529 GG genotype carry a significant age at diagnosis and log PSA correlation despite being different to the TW1 cases by way of median age at diagnosis and proportions of men with high-risk disease. *AKR1C3* is known as an epithelial-mesenchymal transition driver in prostate cancer metastasis^69^. Increased PSA with age at diagnosis associated with the *AKR1C3* rs12529 GG genotype in TW and NZ-non-MPEA cases may indicate that with increasing age, cancers of these men could be progressing. A Japanese study has recorded that those men carrying the *AKR1C3* rs12529 GG genotype have a significantly elevated testosterone level while on ADT and higher cancer progression compared to men carrying the CG and CC genotypes^70^. These authors have further investigated the impact of the *AKR1C3* rs12529 polymorphism on *AKR1C3* enzymatic activity using recombinant proteins. However, they report no variation between the histidine and glutamine variants with *AKR1C3* activity. This may indicate that this SNP is either linked to a different SNP which is functionally associated with *AKR1C3* activity or involved with SNP-SNP interaction leading to this feature. The *AKR1C3* rs12529 polymorphism is known to interact with AR-CAG repeat lengths in increasing prostate cancer-specific mortality while on ADT by up to 13.7 fold^36^, and highly correlated with the *AKR1C3* rs1937845 promoter polymorphism^71^. However, neither the *AKR1C3* rs12529 interaction with the AR-CAG repeat lengths nor the AKR1C3 rs1937845 promoter polymorphism are so far reported as having *AKR1C3* activity associations. In a small group of men with prostate cancer from NZ, we have shown a significant increase in leukocyte *AKR1C3* activity level with age at diagnosis, and upon genetic stratification this shows a trend among cases carrying the *AKR1C3* rs12529 GG genotype only^31^. Additionally, our study also showed that men carrying the *AKR1C3* rs12529 GG genotype carry higher levels of leukocyte AKR1C3 activity, if their PSA was > 20 ng/ml, compared to those carrying a PSA level ≤ 20 ng/ml. It is a possibility that the *AKR1C3* rs12529 GG genotype carriers among NZ cases with an increasing trend of age dependent *AKR1C3* activity could lead to increased ROS production as they age if exposed to more reactive O-quinone formation due to ever-smoking habits. This could result in increased DNA damage in tissue including that of the prostate glandular epithelium with subsequent increases in serum PSA levels. Our previous analysis showed that men carrying the *AKR1C3* rs12529 GG genotype and are ever-smokers are diagnosed at a higher age compared to the same genotype carriers who are never-smokers; and men carrying the *AKR1C3* rs12529 CG genotype and ever-smokers are diagnosed at a higher PSA level compared to the same genotype carriers who are never-smokers^31^.

If the current tested cases and controls cohorts have sufficiently represented the general NZ, US and TW men, the current findings pose the question of validity of the age-based PSA thresholds to be used in prostate cancer screening of all US-EA men or the specific *AKR1C3* rs12529 genotype stratified groups in NZ, US-AA, and TW men. The strength of age and log PSA correlation generally diminishing from controls to cases in all cohorts in univariate model could also mean the irregularity of this correlation with prostate cancer manifestation and progression.

### Study limitations

The relatively small sample sizes from NZ, US-AA, US-EA cohorts diminish statistical power of this analyses. As this study utilized data from previous studies, the consistency of data was limited. The significant variability in demographic, lifestyle, clinical, and prognostic factors between NZ, US and TW cases cohorts limits the strength of the current findings especially on pooled analyses. For example, NZ and US cases consisted of men encompassing the entire spectrum of the disease. However, TW1 and TW2 cases cohorts had distinct clinical differences that may have caused a bias when performing multiple linear regression analyses in pooled cases. Additionally, NZ and US-AA and US-EA cases cohorts with a relatively lower median PSA levels < 10 ng/ml may represent individuals seen either in screening or early detection programs, characteristic of Western Countries. Compared to that, PSA examination based prostate screening is not included in the periodic comprehensive medical examination in Taiwan^72^. Among other deficiencies include the absence of controls among TW men; absence of clinical and prognostic data in a substantial proportion of US and NZ cases; and aspects such as the alcohol consumption data between NZ and US cohorts being not comparable. The absence of tobacco smoking and alcohol consuming lifestyle data from the TW cohorts also added to the restrictions in understanding lifestyle effects among this East-Asian cohort. Concordance between Gleason scores recorded at biopsy and post-RP has shown the superiority of extended biopsies with ≥ 10 cores (median 12 cores), compared to the non-extended biopsies covering < 10 cores (median 6)^73^. However, details on biopsy core number are not accessible to the current analysis and could be a confounding factor. Variation of wait times between PSA testing and diagnosis through biopsy evaluations could also cause confounding effects in the current analysis. However, such information is not available for the current evaluation. The TW cohorts lacked the TNM stage 2 sub-classifications. Therefore, a proportion of cases with T2 stage and having a PSA < 20 ng/ml and a Gleason sum score of < 8 would have wrongfully considered as low risk at prognostic staging.

PSA measurements is a key factor in the current analysis. However, these measurements in cases and controls have been carried out using multiple assay platforms. Such variability can cause variation in recorded PSA level at diagnosis or at recruitment. Stephan et al. have shown a variability ranging from 87 to 115% in various platforms as against a Beckman Coulter (Access) system when considered as the 100% reference^74^. Similarly, measurements made between Abbott Laboratories (Architect i2000) and Roche Diagnostics (Elecsys 2010) methods have shown that the former records 11% less PSA on average^75^. A comparison of VITROS total PSA II and Roche Cobas 8000 e602 has shown an intercept bias of 17%^76^. The authors claim that this is below the desirable specification of inaccuracy of 18.7% suggested by the Westgard Biological Variation Database Specifications. The Westgard database provides a desirable specification for imprecision of 9.1% and a within subject biological variation of 18.1% for PSA^77^. The within subject biological variation is an unavoidable feature while the imprecision due to the use of multiple PSA assay platforms could have incorporated a limitation in our assessment.

## Conclusion

The well-known PSA association with age (age at diagnosis for cases and age at recruitment for controls) was reproduced in combined cross-sectional analyses with multiple linear regression in both cases and controls. However, upon analyses of independent cases cohorts this was reproduced only among NZ-non-MPEA cases. Among controls, PSA was significantly associated with age in all tested cohorts with independent analyses as well as when tested as a pooled group. This indicates that changes have taken place impacting general PSA increase with age upon cancer presentation in some groups. Association of PSA with BMI and tobacco smoking at the expense of age in tested case control cohorts could be indicating a changing paradigm of parameters associated with PSA since this test was established for prostate cancer screening. As the BMI is increasing in most populations with a Western lifestyle, there is a possibility that beyond a median BMI of 27 kg/m^2^ (as reported in the current NZ cases), the ability to be screened with age-based PSA thresholds for prostate cancer could be impaired. Our data suggests that PSA thresholds for prostate cancer screening need refreshing in different ethnicities, in different geographical locations, at different time points while considering genetic variability for its better utility. However, it is too early to know whether the current findings on variable factors affecting PSA outcomes in this cross-sectional analysis are unique only to the current tested cohorts or whether they can be generalized to these ethnicities from different geographical locations. The current findings require further validation with extended cohorts that will provide better statistical power for stratified analyses based on BMI, lifestyle factors and genotype, as well as to reach conclusions that are more robust. Such extended cohorts should be more homogeneous and derived from single localities and for single ethnic groups to provide greater assurance of statistical outcomes. Additionally, using the same features tested from the current case control datasets, machine learning techniques can be attempted to find out reproducibility of the current findings and if possible, produce decision trees utilising these features for better identification of men for prostate biopsies subsequent to PSA-based screening.

## Supplementary Information

Supplementary Tables.

Supplementary Data.

## Data Availability

Data related to this manuscript is available as Supplementary Tables.
