# Supplementary material for: Assessment of factors associated with PSA level in prostate cancer cases and controls from three geographical regions
Source: Sci Rep. 2022 Jan 7;12:55. doi: 10.1038/s41598-021-04116-8 (PMC8742081; doi:10.1038/s41598-021-04116-8)
Supplement: Supplementary file 2 — Supplementary Data. [file 41598_2021_4116_MOESM2_ESM.pdf]

Supplementary results Table 1a. Comparison of prostate cancer cases characteristics from NZ (New Zealand), TW (Taiwan) and US (United States of America).

		**NZ-all (N=515)**	**TW1 (N=645)**	**TW2 (N=643)**	**US-AA (N=489)**	**US-EA (N=487)**	**P value**
Demographic and lifestyle data							
Ethnicity		94.2% NZ-European, 5.8% non-European*	100% Asian	100% Asian	100% US-African	100% US European	-
**BMI (kg/m^2) (median, inter quartile range, N)		27.0 (25.0, 30.0) [512]	[a]24.2 (22.3, 26.2) [377]	[b]24.7 (23.0, 26.4) [379]	27.7 (24.4, 31.2) [488]	27.5 (25.1, 30.3) [487]	<0.001[i]
Tobacco smoking (number and %)	never	226 (44)	NA	NA	136 (28)	188 (39)	< 0.00001[ii] (χ2=29.2)
	ever	287 (56)	NA	NA	353 (72)	299 (61)	
***Alcohol consumption (number and %)	no	149 (29)	NA	NA	71 (15)	50 (10)	< 0.00001[ii] (χ2=65.1)
	yes	366 (71)	NA	NA	418 (85)	437 (90)	
Clinical data							
Age (y) at diagnosis (median, inter quartile range, N)		66 (60, 71) [512]	[c]73 (67, 78) [645]	66 (61,70) [643]	[d]63 (57.3, 68) [488]	65 (60, 71) [490]	<0.001[i]
PSA (ng/ml) at diagnosis (median, inter quartile range, N)		[e]8.6 (5.8, 15.0) [468]	[f]41.0 (15.7, 136) [622]	[g]10.9 (7.0, 18.4) [622]	[h]7.0 (5.2, 12.9) [484]	6.0 (4.6, 9.3) [481]	<0.001[i]
Gleason sum score at diagnosis (median, inter quartile range, N)		[i]7 (6, 7) [515]	[j]7 (6, 8) [632]	[k]7 (7, 7) [643]	7 (6, 7) [202]	6 (6,7) [232]	<0.001[i]
[iii]Prognostic stage group (number and %)	low-risk	253 (49)	89 (14)	455 (71)	69 (34)	106 (46)	< 0.00001[ii] (χ2=443.2)
	high-risk	262 (51)	[l]553 (86)	184 (29)	133 (66)	126 (54)	

*- 3.3%-Maori, Pacific and East Asian (MPEA); 2.5%-Indian sub-continent and Middle-Eastern and others.
**BMI was recorded at enrolment for NZ, US-AA, US-EA, TW1 and TW2 cohorts.
[a]Median BMI of TW1 and TW2 was significantly different to NZ, US-AA and US-EA cohorts
[b]Median BMI of TW1 was significantly different to TW2

***Alcohol consumption among US-AA and US-EA- Those who have ever consumed in their entire life more than 12 alcoholic beverages per year, such as beer, wine, wine coolers or liquor were considered as alcohol consumers.
***Alcohol consumption among NZ was considered as at the study entry, depending on average weekly consumption of one or more alcoholic beverages equivalent to a can of beer, a small glass of wine or a single nip of spirits.
[c]Median age at diagnosis of TW1 cases was significantly different to NZ, TW2, US-EA and US-AA cases
[d]Median age at diagnosis of AA was significantly different to NZ, TW1, TW2 and US-EA cases
[e]Median PSA at diagnosis of NZ cases was significantly different to US-EA cases
[f]Median PSA at diagnosis of TW1 cases was significantly different to NZ, US-EA and US-AA cases
[g]Median PSA at diagnosis of TW2 cases was significantly different to NZ, US-EA and US-AA cases
[h]Median PSA at diagnosis of EA cases was significantly different from US-AA cases
[i,j,k]Median Gleason sum score at diagnosis of NZ, TW1 and TW2 cases was significantly different to US-AA and US-EA cases
[l]Proportion of men in high-risk prognostic stage group in the advanced prostate cancer patients in the TW1 cohort is significantly different to NZ, US-AA and US-EA cases
i - Kruskal-Wallis One Way Analysis of Variance on Ranks test
ii - Chi Square test
[iii]The following criteria were considered in stratifying patients under anatomic prognostic stage group $\geq$IIB (high-risk) - TNM staging $\geq$T2C or PSA $\geq$20ng/ml or Gleason score (GS) $\geq$8 as reported previously [32, 48].

Supplementary results Table 1b. Characteristics of the control study cohorts from NZ and US.

		**NZ-European (N=572)**	**US-AA (N=486)**	**US-EA (N=548)**	**P value**
Demographic and lifestyle data					
BMI (kg/m^2) (median, inter quartile range, N)		26 (24, 29) [547]	29 (26, 33) [486]	27.4 (24.5, 31) [548]	<0.001[i]
Tobacco smoking (number and %)	Never	377 (66)	184 (38)	224 (41)	<0.00001[ii] (χ^2= 104.1)
	Ever	195 (34)	302 (62)	324 (59)	
Alcohol consumption (number and %)	no	80 (14)	107 (22)	69 (13)	<0.0001[ii] (χ^2= 19.6)
	yes	492 (86)	379 (78)	479 (84)	
Age (y) at recruitment (median, inter quartile range, N)		54 (44, 64) [572]	64 (59, 69) [486]	66 (60,73) [548]	<0.001[i]
PSA at recruitment (ng/ml) (median, inter quartile range, N)		0.9 (0.6, 1.9) [498]	0.4 (0.2, 0.8) [412]	0.4 (0.2, 0.8) [476]	<0.001[i]

i- Kruskal-Wallis One Way Analysis of Variance on Ranks test

ii - Chi Square test

Supplementary results Table 2. Comparison of the *AKR1C3* rs12529 genotype frequencies among cohorts

	**CC** n (%)	**CG** n (%)	**GG** n (%)	**G allele %**	**HW equilibrium statistics / P value**
NZ- European controls	181 (40)	202 (44)	71 (16)	38	1.36 P>0.05
NZ- non MPEA cases	121 (33)	162 (44)	83 (23)	45	4.04 P<0.05
NZ-MPEA cases	-	4 (31)	9 (69)	85	-
US-AA cases	58 (29)	105 (52)	39 (19)	45	0.48 P>0.05
US-EA cases	69 (30)	115 (49)	48 (21)	45	0 P>0.05
Taiwan cases-TW1	8 (1)	133 (22)	477 (77)	88	0.14 P>0.05
Taiwan cases-TW2	6 (1)	150 (23)	487 (76)	87	2.27 P>0.05

NZ- non MPEA cases – New Zealanders self-identified as European, or the Indian sub-continent, Middle-East and others
MPEA- Māori, Pacific and East-Asian

The Hardy-Weinberg test statistics is presented for all except for the NZ-MPEA group. Genetic polymorphisms from each cohort except for the NZ-non-MPEA cases cohort was in Hardy-Weinberg equilibrium. There was a significantly higher frequency of cases with the *AKR1C3* rs12529 variant G allele in the NZ-non-MPEA prostate cancer cases cohort compared to the NZ-European non-cancer controls cohort. However, the genotype frequency of the NZ-non-MPEA cases cohort was comparable with US-AA and US-EA cases. TW1 and TW2 cases and the NZ-MPEA cases recorded a higher frequency of the *AKR1C3* rs12529 G allele compared to the rest of the cohorts.

Supplementary results Table 3. Statistical outcomes in the two-way interactive models with smoker*ethnicity and alcohol*ethnicity and the three-way interactions with age at diagnosis*ethnic group*genotype on log PSA outcome for US-EA, US-AA and NZ-non MPEA cases cohorts.

**Source**	**DF**	**Type III SS**	**Mean Square**	**F Value**	**Pr > F**
smoker*ethnicity interaction					
Ethnic Group	2	2.27	1.13	1.39	0.250
Prognostic stage	1	16.28	16.28	19.92	<.0001
Gleason sum	1	37.59	37.59	46	<.0001
Genotype	2	0.64	0.32	0.39	0.675
Age	1	7.25	7.25	8.87	0.003
BMI	1	1.67	1.67	2.04	0.154
Smoker	1	3.98	3.98	4.87	0.028
Alcohol	1	0.66	0.66	0.81	0.368
Smoker*Ethnic Group	2	1.30	0.65	0.79	0.453
alcohol*ethnicity interaction					
Ethnic Group	2	1.160	0.580	0.71	0.492
Prognostic stage	1	16.700	16.700	20.43	<.0001
Gleason sum	1	37.999	37.999	46.49	<.0001
Genotype	2	0.655	0.328	0.4	0.67
Age	1	7.434	7.434	9.09	0.0026
BMI	1	1.879	1.879	2.3	0.130
Smoker	1	3.979	3.979	4.87	0.028
Alcohol	1	0.215	0.215	0.26	0.608
Alcohol*Ethnic Group	2	0.999	0.500	0.61	0.543
Age at diagnosis*Ethnic Group*Genotype interaction					
Ethnic Group	2	3.60	1.80	2.23	0.108
Prognostic stage	1	16.34	16.34	20.28	<.0001
Gleason sum score	1	35.74	35.74	44.34	<.0001
Genotype	2	2.38	1.19	1.47	0.230
Age at diagnosis	1	10.31	10.31	12.79	0.0004
BMI	1	2.11	2.11	2.61	0.106
Smoker	1	4.51	4.51	5.6	0.018
Alcohol	1	0.59	0.59	0.73	0.393
Age at diagnosis*Ethnic Group	2	5.66	2.83	3.51	0.030
Ethnic Group*Genotype	4	5.32	1.33	1.65	0.160
Age at diagnosis*Ethnic Group*Genotype	6	7.93	1.32	1.64	0.133

NZ- non MPEA cases – New Zealanders self-identified as European, or the Indian sub-continent, Middle-East and others
